# Engineering extracellular vesicles for targeted therapeutic delivery in the heart

**DOI:** 10.1042/BSR20254075

**Published:** 2026-04-22

**Authors:** Iqra Anwar, Xinghua Wang, Saverio Parlongo, Sonalí Harris, Richard E. Pratt, Victor J. Dzau, Conrad P. Hodgkinson

**Affiliations:** 1 Mandel Center for Heart and Vascular Research, and the Duke Cardiovascular Research Center, Duke University Medical Center, Durham, NC 27710, U.S.A.; 2 Department of Mechanical and Aerospace Engineering, Politecnico di Torino, Turin 10129, Italy

**Keywords:** Cardiovascular Disease, Extracellular vesicles, Nano-engineering, RNA-based Therapy, Surface functionalization, Targeted Drug Delivery

## Abstract

Heart failure is a leading cause of morbidity and mortality, highlighting the need for improved therapeutic strategies. Critical to the success of therapies is efficient and targeted delivery systems. Extracellular vesicle-based delivery systems have emerged as promising candidates due to their biocompatibility and low immunogenicity. While extracellular vesicles from a wide variety of cells have been used, they have demonstrated divergent effects on the heart. The present review first summarizes the current sources of extracellular vesicles employed in heart failure therapy and their contrasting outcomes. The review then examines the view that these contrasting outcomes arise from limited cell specificity, inefficient delivery, and suboptimal cargo loading. Finally, the review discusses how these problems are being dealt with by recent advances, including genetic modification, chemical functionalization, and enhanced loading strategies. Together, these approaches highlight the potential of extracellular vesicle-based systems as precision therapeutics in cardiovascular medicine.

## Introduction

Heart failure remains a primary cause of mortality worldwide, highlighting the need for more effective therapeutic strategies [1]. Drug delivery to the heart is inefficient, with poor cardiac accumulation and limited cellular specificity [2]. These issues are common in fibrotic or injured myocardial tissue where architectural remodeling and cellular heterogeneity further restrict access [3,4].

One recently emerging candidate for targeted drug delivery is extracellular vesicles. Extracellular vesicles are small vesicles that are secreted from cells to facilitate cell-to-cell communication. As a drug carrier, extracellular vesicles provide various benefits over conventional approaches. Their lipid bilayer structure protects their cargo from degradation in the extracellular environment, ensuring drug stability until delivery to the target cells. Moreover, their endogenous origin makes extracellular vesicles a safer alternative to viral-based approaches [5,6]. These properties make extracellular vesicles particularly well-suited for cardiovascular applications, where biocompatibility and immune evasion are critical for therapeutic success.

Nevertheless, several inherent limitations hamper clinical translation of extracellular vesicle-based therapies. These include rapid clearance and short circulation half-life [7,8], low cargo loading efficiency [9], and insufficient specificity for cell targeting [10]. Furthermore, large-scale production and batch-to-batch consistency remain unresolved [11]. To address these challenges, diverse extracellular vesicle engineering strategies are under active investigation. These include surface functionalization for cell-specific recognition [12,13], active loading techniques to increase encapsulation of therapeutic molecules [14,15], synthetic extracellular vesicles [16], and controlled-release systems [17].

Please note, to be compliant with the recommendations of MISEV2023, the present review will refer to exosomes as extracellular vesicles [18].

## Current applications of extracellular vesicles in the heart

### Extracellular vesicle structure, composition, and function

Various reviews discuss extracellular vesicle structure and biogenesis in depth, and the reader is referred to those [5]. In brief, cell membranes are dynamic and invaginate naturally to form internal vesicles with a wide variety of diameters. Vesicles of ∼30 nm to ∼1 μm in diameter are classified as extracellular vesicles. Extracellular vesicles encapsulate cellular contents such as proteins and RNA molecules. Reflecting the physiological state of their parental cell, the composition of internal cargo is highly heterogeneous. Once formed, extracellular vesicles are secreted into the extracellular space. Passive movement in the extracellular space leads to interactions between extracellular vesicle membrane proteins and membrane proteins in neighboring cells. These interactions promote extracellular vesicle internalization. In essence, the process of extracellular vesicle secretion and internalization enables cells to communicate their physiological states to each other [19].

### Native, engineered, and biomimetic extracellular vesicles

Extracellular vesicles used for therapeutic applications can be classified into three groups: native, engineered, and biomimetic (or synthetic) vesicles. The advantages and disadvantages of each group are described in Table 1.

**Table 1:** Comparison of native, engineered, and biomimetic extracellular vesicles

Feature	Native extracellular vesicles	Engineered extracellular vesicles	Biomimetic /synthetic extracellular vesicles
Origin	Naturally secreted by cells	Native extracellular vesicles modified by genetic or chemical engineering	Constructed artificially to mimic extracellular vesicle structure and function
Stability	High physiological stability; optimized by evolution	Stability can be enhanced (e.g., PEGylation), but modifications may impact membrane integrity	Generally high due to optimized composition, but long-term behavior *in vivo* is less understood
Targeting efficiency	Intrinsic tropism depends on parental cell type	Strongest targeting capability via surface engineering (ligands, peptides)	Tunable targeting possible, but clinical targeting data still limited
Biocompatibility/immunogenicity	Excellent biocompatibility; low immunogenicity	Biocompatible but engineering steps may introduce toxicity or immune activation	Composition control can ensure compatibility; risk of immune response if non-biological materials used
Cargo loading	Limited loading control; cargo is cell-dependent	Enhanced and specific loading of RNAs, drugs, or proteins	Very high loading capacity depending on design
Scalability	Challenging—low yield and heterogeneity	More complex and costly to scale	Best scalability—standardizable manufacturing possible
Regulatory path	Hard to standardize due to biological variability	Need to justify engineered modifications	Potentially simpler if components are well-defined and GMP-compatible
Strengths	Natural targeting + safety	Precision therapeutic delivery	Defined composition + scalable
Limitations	Low yield, heterogeneity, limited targeting	Risk of structural alteration, toxicity, high cost	Unproven long-term biocompatibility, may lack natural cues

Native extracellular vesicles are naturally secreted by cells, giving them inherently high biocompatibility and minimal immunogenicity. These are clear advantages for therapeutic applications. However, their therapeutic efficacy can be limited by low yield and significant heterogeneity. Moreover, natural extracellular vesicles may carry unwanted biological cargos.

Engineered extracellular vesicles are native vesicles that have been modified to enhance therapeutic performance. Genetic and chemical engineering approaches have been employed to enrich extracellular vesicles with specific RNAs, proteins, or small molecules. Similarly, surface engineering has been used to add targeting ligands for cell-specific delivery. However, engineering may impair structural integrity as well as increasing manufacturing complexity with higher production costs.

Biomimetic or synthetic extracellular vesicles represent a third approach. In this approach, extracellular vesicles are built from the ground up. The objective is to recapitulate key structural and functional features of natural extracellular vesicles while offering superior control over composition. These biomimetic extracellular vesicles can provide high loading capacity, enhanced stability, and improved scalability when compared with biologically derived vesicles. However, despite their design flexibility, biomimetic systems currently lack the full repertoire of biological ‘cues’ present in native extracellular vesicles. Moreover, their immunological and pharmacokinetic behavior *in vivo* needs to be clarified.

In the following three sections, we discuss how native, engineered, and biomimetic extracellular vesicles are applied for heart therapy.

### Native extracellular vesicles

In the context of heart therapy, native extracellular vesicles have been isolated from a plethora of cardiac and circulating cells. Their appeal lies in the presence of cargo molecules that can promote repair and regeneration. However, these extracellular vesicles often contain deleterious components. In the following section, we review the major cellular sources of native extracellular vesicles as well as the often-conflicting biological activities of their cargoes.

#### Cardiomyocyte-derived extracellular vesicles

Cardiomyocytes are the main constituent of the heart. As muscle cells, they enable the heart to pump blood. Cardiac injury results in cardiomyocyte cell death. The human heart lacks any meaningful regenerative capacity; therefore, the loss of cardiomyocytes is permanent. The permanent loss of cardiomyocytes results in the heart having to work harder. The extra effort sets up a deleterious feedback loop whereby the heart eventually fails.

**Figure 1:**
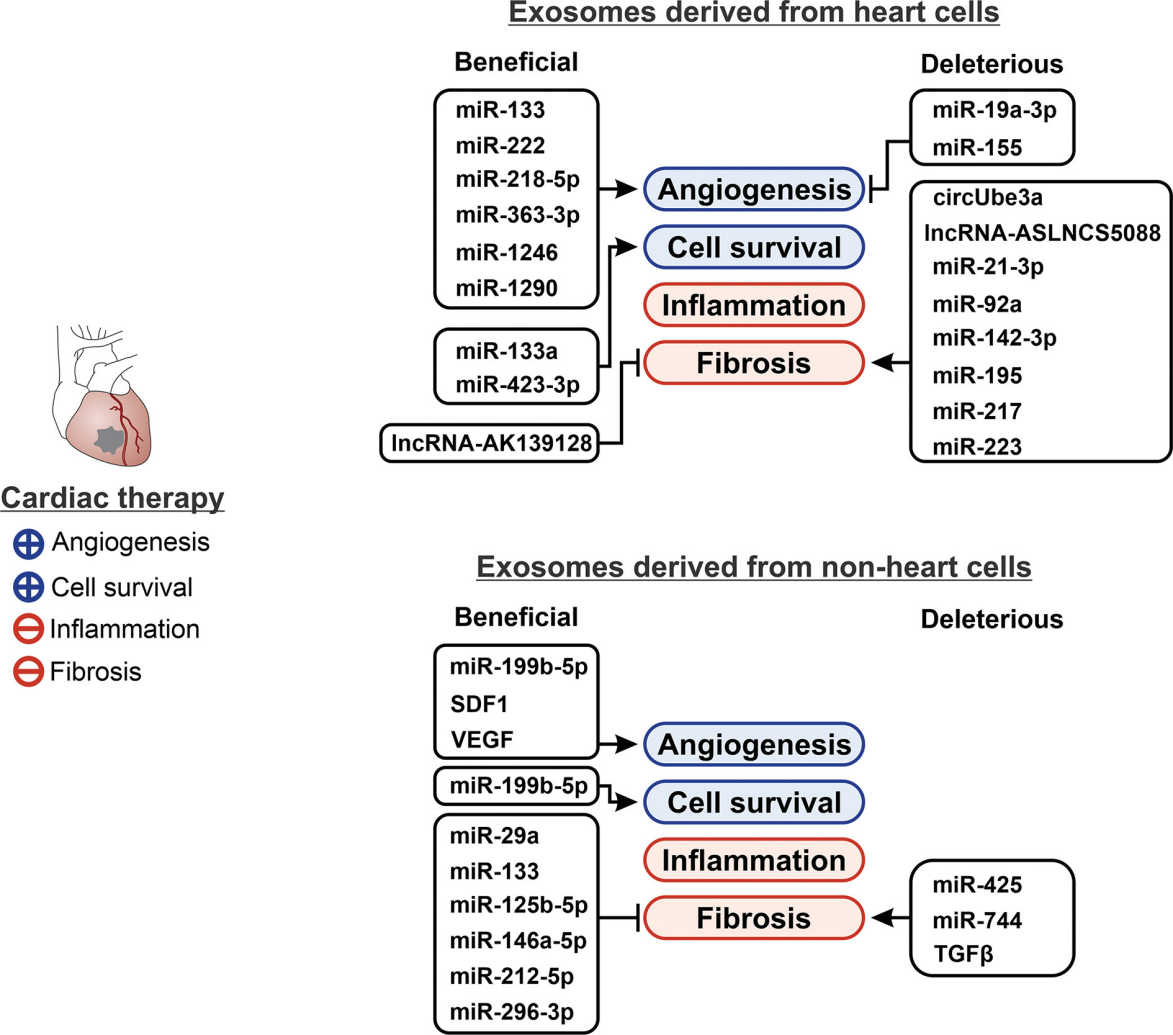
Extracellular vesicles have confounding effects on the heart An ideal cardiac therapy would improve cell survival, promote revascularization, dampen inflammation, and reduce fibrosis. Unfortunately, contents of extracellular vesicles, whether derived from heart or non-heart sources, tend to have conflicting effects on cell survival, vascularization (angiogenesis), and fibrosis. This figure summarizes identification of important extracellular vesicle mediators and their effects on key therapeutic processes.

Cardiomyocytes in the failing heart have recently been identified as a source of extracellular vesicles. These extracellular vesicles have been shown to influence both angiogenesis and fibrosis. However, the effects are complex, with the contents of cardiomyocyte-derived extracellular vesicles often counteracting each other (Figure 1).

Components of cardiomyocyte-derived extracellular vesicles that are believed to influence angiogenesis include miR-19a-3p and miR-222. The levels of both miRs are increased in cardiomyocyte-derived extracellular vesicles following ischemia. MiR-222 plays a positive role, promoting endothelial cell proliferation and angiogenesis [20]. In contrast, by targeting hypoxia inducible factor-1α (HIF-1α), miR-19a-3p inhibits endothelial cell proliferation and prevents angiogenesis [21].

A similar duality is seen with respect to fibrosis. For example, cardiomyocyte-derived extracellular vesicles are enriched in several miRs that promote fibrosis: miR-92a, miR-195, and miR-217. Fibroblast proliferation is enhanced by miR-217 modulating PTEN activity [22]. Furthermore, proliferative fibroblasts are aided in their differentiation to myofibroblasts by miR-92a and miR-195. This occurs in part through down-regulation of the α-smooth muscle actin (α-SMA) repressor Smad7 [23]. However, vesicular lncRNA AK139128 suppresses fibroblast growth and slows down fibrosis [24].

#### Fibroblast-derived extracellular vesicles

Fibroblasts play an important role in maintaining the extracellular matrix (ECM). Their numbers significantly increase following cardiac injury and during heart failure. Proliferative signals also promote their differentiation into myofibroblasts, where they begin to secrete copious amounts of matrix proteins. Deposition of such a large amount of ECM material stiffens the heart. Heart stiffening impairs contractile performance.

Fibroblast-derived extracellular vesicles have dual, opposing effects on the failing heart depending on the internalizing cell (Figure 1).

Internalization of fibroblast-derived extracellular vesicles by cardiomyocytes is complex. In one study, fibroblast-derived extracellular vesicles were shown to improve cardiac performance by increasing expression of the gap junction protein connexin-43 in cardiomyocytes [25]. Similarly, ischemia increases levels of the cardioprotective miR, miR-133a, in fibroblast-derived extracellular vesicles. MiR-133a prevents cardiomyocyte apoptosis by reducing expression of embryonic lethal abnormal vision-like 1 protein [26]. Cardiomyocyte apoptosis is also reduced by the fibroblast-derived vesicular constituent miR-423-3p. In this case, apoptosis is reduced via the targeting of the Ras-related protein Rap-2c [27]. In contrast, fibroblast-derived extracellular vesicles can also promote cardiomyocyte hypertrophy by delivering miR-21-3p.

Similarly, re-internalization of fibroblast-derived extracellular vesicles by fibroblasts is generally deleterious. Cardiac fibroblasts contain miR-223, which promotes fibroblast proliferation and differentiation [28,29]. While miR-223 has not been reported to be present in cardiac fibroblast extracellular vesicles, the miR has been shown to be present in extracellular vesicles derived from cancer-associated fibroblasts [30].

#### Endothelial-derived extracellular vesicles

In response to injury, endothelial progenitor cells (EPCs) are recruited from the bone marrow to the heart to help revascularize the tissue. This process is facilitated by extracellular vesicles released from EPCs. EPC-derived extracellular vesicles redirect fibroblast differentiation away from a myofibroblast fate toward a vascular fate by up-regulating expression of endothelial-specific markers (vascular endothelial growth factor receptor-2) and down-regulating myofibroblast markers (α-SMA, collagen I, and TGF-β) [31]. Similarly, ischemia increases levels of the angiogenic miRs miR-133, miR-218-5p, miR-363-3p, miR-1246, and miR-1290 in EPC-derived extracellular vesicles. MiR-133 targets YBX-1 and promotes angiogenesis through the mesenchymal-endothelial transition [32]. The mesenchymal-endothelial transition is also enhanced by miR-218-5p and miR-363-3p (p53/JMY signaling pathway) as well as by miR-1246 and miR-1290 (E74-like factor-5 and specificity protein-1) [33,34] (Figure 1).

#### Immune cell-derived extracellular vesicles

Cardiac injury and aging, both drivers of heart failure, lead to the accumulation of immune cells in the heart. Extracellular vesicles released from immune cells influence the heart both positively and negatively (Figure 1).

Extracellular vesicles derived from inflammatory-phenotype (M1) macrophages are enriched in miR-155. Once internalized by endothelial cells, miR-155 disrupts vascularization by targeting key angiogenic genes, including small GTPase rac1 (RAC1), p21-activated kinase 2, and Sirtuin 1 [35].

Other immune cell-derived extracellular vesicles appear to promote fibrosis. Extracellular vesicles derived from reparative-phenotype (M2) macrophages promote fibrosis by delivering lncRNA-ASLNCS5088 and circUbe3a into fibroblasts. The lncRNA promotes collagen synthesis by sequestering miR-200c-3p [36], while circUbe3a promotes fibroblast differentiation into myofibroblasts by targeting RhoC [37]. Similarly, extracellular vesicles from activated CD4+ T cells also promote fibroblast differentiation into myofibroblasts by delivering miR-142-3p. This miR enhances WNT signaling [38].

Extracellular vesicles from M2 macrophages can also be beneficial since they prevent cardiomyocyte apoptosis. These extracellular vesicles deliver miR-1271-5p into cardiomyocytes, and the miR prevents cell death by targeting SOX6 [39].

#### ECM-associated extracellular vesicles

Extracellular matrix-bound extracellular vesicles (MBVs) represent a distinct subpopulation that differs in meaningful ways from secreted extracellular vesicles. Although both originate from cells, MBVs remain physically embedded within the fibrillar ECM rather than being released into interstitial fluid or circulation. This tethering to the matrix gives MBVs a unique spatial distribution and release profile: they are only liberated when the ECM undergoes remodeling, degradation, or mechanical deformation. In contrast, classical extracellular vesicles are secreted directly into the fluid phase, diffuse through tissues, and can act locally or systemically.

MBVs also exhibit compositional differences. Comparative studies show that when compared with extracellular vesicles recovered from culture media or bodily fluids, MBVs carry distinct protein and miRNA signatures. MBV cargo is typically enriched in molecules involved in ECM organization, proteolysis, immune modulation, and local tissue repair. This compositional difference implies distinct biological roles. Secreted extracellular vesicles function as mobile intercellular messengers, delivering cargo to distant or nearby recipient cells. In contrast, MBVs appear to act as localized signaling reservoirs that influence only those cells in direct contact with the ECM. Their sequestration within the matrix confers high stability and enables sustained, spatially restricted delivery of bioactive cues as the ECM naturally remodels.

Although MBVs have not been fully characterized in the heart, their potential relevance is high. For example, cardiac remodeling after injury is dominated by ECM turnover, a key process that MBVs could influence [40–43].

The unique sequestration of MBVs within the ECM offers distinct therapeutic advantages over circulating extracellular vesicles. Because they are physically tethered to the matrix, MBVs are protected from rapid systemic clearance and enzymatic degradation, providing a localized and sustained signaling reservoir that is critical for tissue remodeling [42]. Furthermore, their cargo is highly tissue-specific; for instance, MBVs derived from the cardiac ECM contain unique miRNA profiles—distinct from plasma extracellular vesicles—that are specifically tuned to ameliorate fibrosis and support mitochondrial function [43].

However, significant limitations remain regarding their translation. The primary challenge lies in the isolation process, which requires enzymatic digestion of the host tissue or scaffold. This process is not only more labor-intensive than biofluid isolation but also carries the risk of altering the vesicle surface markers or introducing matrix contaminants. Additionally, while MBVs show promise for integration into cardiac patches, their standard characterization is less mature than that of exosomes, making large-scale good manufacturing practice (GMP) manufacturing and quality control more complex.

#### Circulating extracellular vesicles

Extracellular vesicles from nearly all cell types exist in the circulation. These extracellular vesicles have also been shown to have both positive and negative effects on cardiac disease (Figure 1). For example, circulating extracellular vesicles have been reported to both activate and inhibit fibrosis. Circulating extracellular vesicles contain the profibrotic miR, miR-21 [44]. Similarly, circulating extracellular vesicles from heart failure patients are depleted in miR-425 and miR-744, which appears to promote fibrosis by enabling TGF-β1 signaling [45]. In contrast, circulating extracellular vesicles are also a source of anti-fibrotic miRs such as miR-29a and miR-133 [46].

#### Mesenchymal stem cell-derived extracellular vesicles

Mesenchymal stem cells (MSCs) are multipotent adult stem cells that can differentiate into bone, cartilage, and fat cells. Injected MSCs improve cardiac function in the injured heart through a paracrine mechanism. Consequently, many researchers have investigated using MSC-derived extracellular vesicles as an alternative to the cells themselves (Figure 1).

MSCs are well known for their ability to prevent fibrosis via paracrine actions of secreted proteins as well as through release of extracellular vesicles. High levels of various anti-fibrotic miRs are found in MSC-derived extracellular vesicles. One such example is miR-29b-3p. Following cardiac injury, levels of this miR are reduced in the myocardium. Delivery of miR-29b-3p into the myocardium by MSC-derived extracellular vesicles inhibits fibrosis by reducing the expression of the proteins ADAMTS16, MMP2, and MMP9 [47,48]. A similar situation exists with miR-212-5p. Expression of this miR is reduced by cardiac injury, while it is abundant in MSC-derived extracellular vesicles. MiR-212-5p from MSC-derived extracellular vesicles prevents fibrosis by targeting VEGF and TGF-β1 pathways [49]. Moreover, MSC-derived extracellular vesicles are a source of miR-125b-5p. This miR reduces expression of the pro-fibrotic protein Smad7 [50].

#### Cardiosphere-derived extracellular vesicles

Cardiospheres are self-assembling, three-dimensional clusters of stem cells and other supporting cells that have regenerative potential. Their mode of action has been ascribed to extracellular vesicles, notably increasing levels of pro-angiogenic proteins (SDF1 and VEGF) and anti-fibrotic miRs (miR-146a-5p) in recipient cells [51,52] (Figure 1).

#### Human induced pluripotent stem cell-derived extracellular vesicles

Human induced pluripotent stem cells (hiPSCs) are generated via the overexpression of Yamanaka factors in fibroblasts. hiPSCs are totipotent and can potentially be differentiated into any cell type. Extracellular vesicles derived from hiPSCs promote cardiomyocyte survival [53] and promote angiogenesis by delivering miR-199b-5p [54]. In contrast, hiPSC-derived extracellular vesicles could potentially be pro-fibrotic as they promote fibroblast proliferation [55] (Figure 1).

#### Cortical bone stem cell-derived extracellular vesicles

Cortical bone stem cells are another potential source of anti-fibrotic extracellular vesicles. In one study, extracellular vesicles derived from these cells were demonstrated to have anti-fibrotic properties by reducing the expression of a small nucleolar RNA (snoRNA) involved in stabilizing ribosomes [56].

### Engineered extracellular vesicles

As described in the previous section, native extracellular vesicles can have conflicting effects on the heart. For therapies, it is important to retain the benefits of extracellular vesicles while removing their confounding effects. For many researchers, the approach taken has been to engineer extracellular vesicles. The following sections describe these extracellular vesicle engineering strategies in more detail.

#### Engineering extracellular vesicle loading

Issues with extracellular vesicle loading add another layer of complexity to the challenges of cell-specificity and sustained delivery. Here, the issue is consistently loading a potent therapeutic cargo into extracellular vesicles. The key challenges are loading variability, inefficient encapsulation of therapeutic materials, extracellular vesicle integrity, and cargo-induced toxicity.

Extracellular vesicle loading methods vary widely in their effectiveness. Passive methods, such as incubation, are very simple but yield low efficiency [57–59]. Passive loading methods can be improved by modifying the therapeutic agent to improve translocation across the membrane. For example, by modifying nucleic acids with cholesterol [58,60]. Active methods, such as electroporation, sonication, and transfection, are more efficient. Transfection can also take the form of chimeric extracellular vesicle–liposome hybrids to improve encapsulation and scalability [61–63]. Active loading methods risk damaging the extracellular vesicle [59,60,64–67]. Damaged extracellular vesicles can lose their cargo prematurely or trigger an immune response. The latter is especially problematic because it can lead to a harmful inflammatory reaction in the heart, counteracting the intended therapeutic effect. Moreover, the extracellular vesicle membrane also works against efficient loading. Large molecules like CRISPR-Cas9 systems and nucleic acids do not effectively cross the plasma membrane. Similarly, the intrinsically hydrophobic nature of a lipid bilayer makes it difficult to load hydrophilic molecules. Poor loading means the therapeutic benefit of a cargo is overwhelmed by background noise, giving rise to a negligible effect.

A separate issue with extracellular vesicle loading is cargo-induced toxicity. In addition, extracellular vesicles encapsulate cellular contents, which, by their nature, are highly diverse. As discussed above, such diversity means that encapsulated material may include agents that may actively work against the therapeutic agent. Methods exist to remove extracellular vesicle contents; however, they can disrupt extracellular vesicle integrity. An alternative approach is parental cell engineering. Here, gene-editing machinery is employed to remove deleterious material. The added advantage of such an approach is that it is also possible to modify the cells to express the therapeutic agent. However, while parental cell engineering offers high specificity, it can be complex and may affect the biology of parent cells.

While efficient cargo loading remains a challenge across all extracellular therapies, it is particularly critical for cardiovascular applications where precise dosing is required to overcome the high-clearance environment of the heart. For instance, passive loading of cholesterol-conjugated miRNAs (e.g., miR-21 or miR-146a) has been successfully employed to enhance the anti-fibrotic potency of extracellular vesicles, allowing for higher therapeutic concentrations within the ischemic niche [68].

To deliver larger or more complex payloads, such as mRNAs (e.g., VEGF) or CRISPR/Cas9 components for gene editing (e.g., phospholamban gene in heart failure), active loading via electroporation or the use of extracellular vesicle–liposome hybrids is required. These hybrid systems combine the high loading capacity of synthetic lipids with the natural homing abilities of the extracellular vesicle membrane, specifically facilitating the delivery of pro-angiogenic factors to hypoxic cardiomyocytes while minimizing the risk of systemic cargo-induced toxicity [69–71]. Furthermore, parental cell engineering of MSCs to overexpress GATA-4 has been shown to naturally enrich the resulting extracellular vesicles with cardioprotective signals, bypassing the integrity issues associated with *ex vivo* loading methods [72].

#### Engineering strategies for cell-specific binding

**Figure 2:**
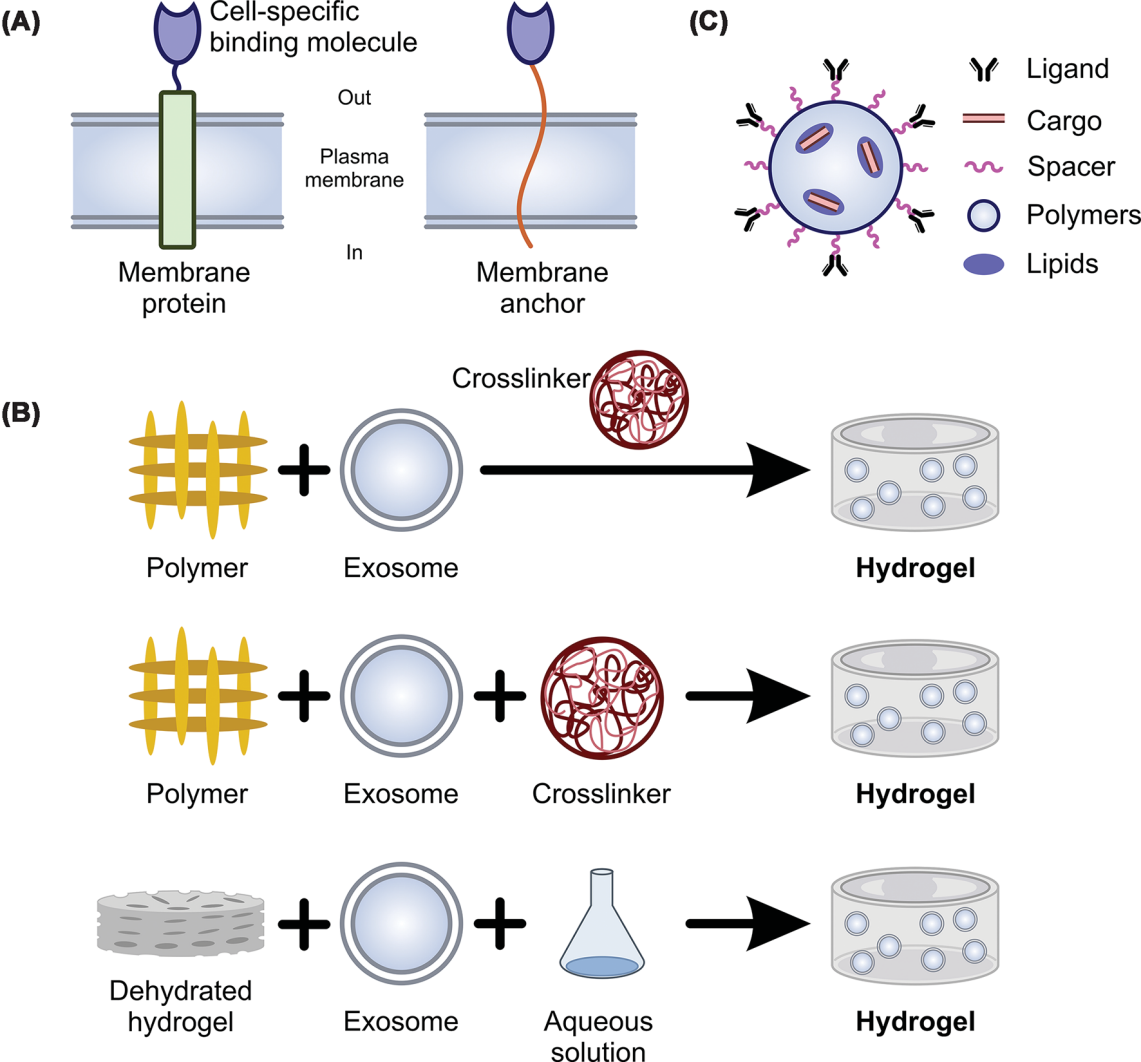
Extracellular vesicle engineering To improve the utility of extracellular vesicles, researchers have focused on cell-specificity, sustained release, and improved loading. (**A**) Several approaches are used to generate cell-specific extracellular vesicles. One approach involves coupling a cell-specific binding molecule to an extracellular vesicle membrane protein. Examples include LAMP2, PDGFR, and Tetraspanins. Coupling is achieved either genetically or through chemical modification (e.g., click chemistry). Cell-specific binding molecules are either peptides or single-chain variable fragment antibodies. (**B**) Sustained release is typically achieved through hydrogels. Encapsulation of extracellular vesicles into hydrogels is achieved in one of three ways. Firstly, polymers and extracellular vesicles are incubated together, and the hydrogel is generated via the later addition of cross-linkers. A related approach is to mix polymers, extracellular vesicles, and cross-linkers together. The last approach involves incubating a dehydrated hydrogel with extracellular vesicles in an aqueous solution. Re-hydration of the hydrogel pulls in the extracellular vesicles. (**C**) Improved loading takes many forms. However, many researchers are taking the approach of synthetic extracellular vesicles. Synthetic extracellular vesicles have the highest loading efficiency, defined contents, and are highly adaptable. The figure shows a schematic of the structure of a common synthetic extracellular vesicle, one that is functionalized with a cell-specific antibody.

Extracellular vesicles are generally not cell-specific. This is problematic because, as outlined above, the recipient cell internalizing the extracellular vesicle often determines whether the extracellular vesicle is beneficial or not. Thus, one important way to improve extracellular vesicle-based therapies is to engineer cell specificity (Figure 2A).

Fortunately, extracellular vesicle membranes can be readily engineered to enhance functionality. Engineering for cell-specificity is primarily achieved through genetic engineering or chemical modification [73,74]. In genetic approaches, the gene encoding a targeting peptide or protein is fused with that of a selected extracellular vesicle membrane protein. Donor cells transfected with plasmids encoding these fusion constructs release engineered extracellular vesicles that display the desired targeting ligands on their surface. One of the most used extracellular vesicle proteins for this purpose is lysosome-associated membrane protein 2B (LAMP-2B). The N-terminal domain is accessible on the extracellular vesicle surface, making it suitable for attaching targeting sequences [73,75]. Cell-specific binding peptides or single-chain antibody fragments have been identified and their DNA sequences genetically fused to the N-terminus of LAMP-2B. Peptides generally exhibit modest binding affinities. In contrast, antibodies or affibodies typically show much higher affinities with binding coefficients often in the low nanomolar range for their respective targets [76]. A prominent cardiovascular application of this (LAMP-2B) approach involves the use of cardiac targeting peptide (CTP). Researchers genetically fused the CTP sequence (APWHLSSQYSRT) to the N-terminus of LAMP-2B in HEK293 cells, resulting in extracellular vesicles that demonstrated significantly enhanced uptake by cardiomyocytes *in vitro* and increased accumulation in heart tissue *in vivo* following intravenous injection [77].

The transmembrane domain of platelet-derived growth factor receptor (PDGFR) has also been used as a substrate for engineering cell specificity. The PDGFR is commonly found in the extracellular vesicle membrane. In one study, the sequence for the epidermal growth factor receptor (EGFR)-binding peptide GE11 was genetically fused to the PDGFR transmembrane domain to generate GE11-bearing extracellular vesicles. Similarly, engineering for ischemia-specific binding has been achieved by displaying ischemic myocardium-targeting peptide on the extracellular vesicle surface. These engineered vesicles show a high affinity for hypoxic cardiomyocytes, effectively delivering therapeutic payloads like miR-21 to the infarcted region to reduce apoptosis and improve cardiac function [78].

Beyond LAMP-2B and PDGFR, tetraspanin proteins are also an attractive substrate. Tetraspanins, such as CD63, CD9, and CD81, are commonly found in the membranes of a wide variety of extracellular vesicles. Importantly, tetraspanins contain two extracellular loops (EC1 and EC2) that can be engineered to display targeting sequences. While therapeutic uses have yet to be explored, the fluorescent protein pHluorin was inserted into the small extracellular loop (EC1) to visualize extracellular vesicle secretion and uptake [79].

In addition to coupling cell-specific binding moieties to extracellular vesicle membrane proteins, cell-specific binding agents can be attached directly to the extracellular vesicle membranes via anchors. Two membrane anchors are commonly employed: glycoinositol phospholipids (GPIs) and palmitoylation peptides. GPIs act as membrane anchors by embedding their lipid tails into the outer leaflet of the membrane [80]. GPI-anchored EGFR-specific nanobodies have been used to effectively target EGFR-positive A431 cells [81]. Proteins with a GPI anchor are incorporated into the extracellular vesicle membrane via simple incubation. In contrast, proteins with a palmitoylation peptide anchor must be expressed in the cells providing the extracellular vesicles. This is because the palmitoylation peptide is not a membrane anchor per se; rather, it is the substrate for cellular enzymes to generate the membrane linker palmitic acid. The palmitoylation signal peptide MLCCMRRTKQ has been demonstrated to effectively label extracellular vesicles with fluorescent proteins [82]. While both GPI and palmitoylation anchors can label extracellular vesicle membrane, with cell-specific binding moieties, there are differences between the two anchors that have therapeutic implications. First, the GPI molecule is larger and more complex to generate when compared with a palmitoylation peptide. Perhaps more importantly, GPI anchoring is irreversible while palmitoylation anchors are reversible. Irreversible anchors are ideal for applications requiring long-term, sustained engagement with the cell. In contrast, reversible anchors provide an inherent ‘Off’ switch and enable more precise fine-tuning of drug delivery.

Chemical conjugation is another approach to engineer the surface of extracellular vesicles. Click chemistry, particularly azide–alkyne cycloaddition, is a widely used method. This reaction enables the formation of a stable triazole linkage between an azide and an alkyne moiety, allowing for selective and covalent attachment of peptides, antibodies, fluorescent dyes, or radiotracers to extracellular vesicle membranes [83–86]. The traditional copper-catalyzed variant of this reaction (CuAAC) poses toxicity concerns due to the presence of copper ions [87]. To address this, strain-promoted azide–alkyne cycloaddition (SPAAC) has been developed as a copper-free alternative. SPAAC utilizes strained alkynes such as cyclooctynes to react efficiently with azides under mild, physiological conditions [88–90]. An alternative to both CuAAC and SPAAC is provided by bifunctional chelators (BFCs). BFCs contain a functional group (amines, thiols, or carboxyls) for covalent attachment to extracellular vesicle membranes [91].

In the context of chemical modification, click chemistry has been used to functionalize EV surfaces with platelet-derived membranes or ‘homing’ antibodies. This creates platelet-mimetic extracellular vesicles that naturally gravitate toward injured vascular endothelium and sites of myocardial infarction, enhancing the local concentration of delivered therapeutics [92].

Beyond click chemistry, amine cross-linking and maleimide–thiol coupling are also widely used for extracellular vesicle modification. Amine cross-linking targets surface amine groups, while maleimide-thiol reactions form stable bonds between thiol-containing molecules and maleimide-functionalized extracellular vesicles [93–95]. These methods enable hydrophobic molecules to integrate directly into the lipid bilayer [93,96]. This approach is less selective but is advantageous when functional groups are limited or unavailable on extracellular vesicle membranes.

#### Engineering sustained delivery

Even when extracellular vesicles successfully reach their target cell type in the heart, their therapeutic effect is often short-lived. Systemic injection of extracellular vesicles results in their rapid clearance from the bloodstream by macrophages and other cells of the mononuclear phagocyte system. The plasma half-life of extracellular vesicles is often very short, sometimes as little as 70–80 min. Similarly, due to rapid blood flow through the heart, the extracellular vesicles that do arrive may not stay long enough to deliver a meaningful and prolonged therapeutic payload. This means that the therapeutic effect is transient, and repeated doses may be required, which complicates clinical applications. Heart failure is a chronic condition characterized by continuous, adverse remodeling of cardiac tissue. A therapeutic effect that is not sustained over a long period will likely fail to counteract these ongoing pathological changes. To that end, there has been a focus on how to sustain extracellular vesicle delivery.

Hydrogels have emerged as a promising candidate due to their biocompatibility, tunable degradation, and ability to provide localized, sustained release [97]. In essence, hydrogels are comprise hydrophilic polymers arranged in a three-dimensional network with a large water content. Polymers for hydrogels can be natural or synthetic. Natural polymers are typically ECM proteins such as collagen, gelatin, or hyaluronic acid, while synthetic polymers include PEG (polyethylene glycol), polylactic-co-glycolic acid, or polyhydroxyethyl methacrylate. By adjusting the composition of the hydrogel, hydrogels are highly tunable. Composition is particularly important for modifying the degradation rate of the hydrogel. Through modifications of the degradation rate it is possible to fine-tune the rate at which entrapped extracellular vesicles are released [98]. Extracellular vesicle encapsulation is carried out in one of three ways. One method involves initially mixing the extracellular vesicles with the polymers. Once thoroughly mixed, cross-linkers are added to make the hydrogel. The second method is a variant, whereby extracellular vesicles, polymers, and cross-linkers are incubated together. The final method introduces extracellular vesicles after hydrogel polymerization. Here, water is initially removed from the hydrogel, followed by soaking in an aqueous solution containing extracellular vesicles. In response to soaking, the hydrogel swells and ‘breathes in’ the extracellular vesicles. Once the hydrogel is in place, the extracellular vesicles essentially leak out at a rate dependent on pore size [99–101] (Figure 2B). Hydrogels composed of natural ECM proteins, such as collagen or hyaluronic acid, are particularly suited for the myocardium, as they can be engineered to match the mechanical stiffness of cardiac tissue, thereby supporting structural integrity while providing localized, sustained extracellular vesicle release [102].

More recently, bioprinting has evolved as a novel method for making hydrogels. The advantage of bioprinting is the ability to more closely match the host ECM [103]. Moreover, hydrogel alternatives include decellularized cardiac scaffolds. Seeding such scaffolds with cATMSC-derived extracellular vesicles reduced inflammation and promoted angiogenesis in a myocardial infarction model [104,105].

### Biomimetic extracellular vesicles

To address the limitations surrounding native and engineered extracellular vesicles, researchers are developing biomimetic extracellular vesicles [106,107]. Biomimetic extracellular vesicles are synthetic nanovesicles designed to recapitulate the structural and functional properties of natural extracellular vesicles. Unlike natural extracellular vesicles, biomimetic synthetic vesicles can be produced in large quantities with a composition tailored for their intended therapeutic purpose. Fabrication strategies follow either a ‘top-down’ or ‘bottom-up’ approach.

#### Top-down

Early efforts to construct biomimetic extracellular vesicles utilized the natural ability of cells to generate vesicles with defined protein–lipid compositions. The approach taken was called top-down in that cells were disrupted down into nanoscale vesicles. Once cells were disrupted into membrane sheets, these sheets would reassemble into vesicles that retained many features of natural extracellular vesicles, such as membrane proteins and lipid composition, while showing far less heterogeneity. The cell-derived starting point created biomimetic vesicles with complex biological surfaces but without the unpredictable cargo typical of native extracellular vesicles. From this foundation several fabrication approaches arose, with each approach trying to perfect membrane reorganization into stable vesicles of nanoscale size [108].

Extrusion-based strategies provided the earliest and simplest solutions. When cells were forced through polycarbonate membranes of successively smaller pore sizes, the resulting nanoscale vesicles were remarkably uniform. This uniformity, which natural extracellular vesicles do not have, enables large-scale production and reliable drug loading. These extrusion methods also demonstrated that vesicles retaining native membrane proteins could achieve targeted drug delivery, although their scalability and delivery efficiency remained practical challenges [109,110].

Scaling extrusion methods often compromise size uniformity and reproducibility, leading to quality control issues across batches. Thus, sonication-based techniques were developed as an alternative. In these approaches, cells are first fragmented under alkaline conditions to release cytoplasmic material. Once cytoplasmic material is removed, the purified membrane sheets are sonicated into new vesicles [111,112].

Extrusion- and sonication-based methods can negatively influence membrane protein function. The need to preserve membrane protein function led to the development of nitrogen-cavitation–based methods. In this approach, cells are first exposed to high-pressure nitrogen, which dissolves into the cytoplasm. Upon decompression, the dissolved nitrogen rapidly expands and fragments the cell. Unlike mechanical disruption methods described above, decompression ruptures the cell without extensively denaturing or stripping surface proteins. With membrane proteins remaining correctly oriented and functionally intact, the resulting vesicles retain native signaling, adhesion, and targeting capabilities. At the same time, nitrogen cavitation produces nanosized vesicles at yields far exceeding natural extracellular vesicle secretion. This combination, high yield plus preserved biological functionality, made nitrogen-derived vesicles an important step toward scalable, cell-derived, therapeutic, biomimetic extracellular vesicles [111,113].

Finally, chemical or mechanically induced blebbing repurposed a natural cellular process as a production method. When cells were exposed to osmotic or chemical stress, they formed nanoscale membrane blebs that could be harvested as vesicles. This approach solved the problem of inconsistent vesicle size in earlier blebbing methods by inducing more uniform nanoscale blebs [114].

Across all methods, the field has progressed from the general challenge of breaking cells into membrane fragments to the more specific challenge of preserving, removing, or exploiting particular biological components. Each strategy, whether it be extrusion, sonication, nitrogen cavitation, or controlled blebbing, offers a distinct balance between biological fidelity, production yield, and cargo control. Together, these approaches demonstrate the promise of top-down cell-derived vesicles as scalable and customizable extracellular vesicle mimetics for therapeutic use.

#### Bottom-down

Alternatives to ‘top-down’ are hybrid and ‘bottom-up’ methods. Hybrid extracellular vesicles are produced by fusing natural extracellular vesicles with synthetic nanoparticles such as liposomes. In essence, the biocompatibility of natural extracellular vesicles is augmented with the stability and modularity of synthetic carriers. In contrast, ‘bottom-up’ strategies construct extracellular vesicles *de novo* from defined components such as lipids, proteins, or peptides. Advances in synthetic biology have enabled the rational design of extracellular vesicles with programmable surface features and cargo profiles, offering greater control over targeting and function than either natural or top-down-derived extracellular vesicles [108] (Figure 2C).

The bottom-up strategy uses simple molecular components to generate vesicles that replicate selected features of natural extracellular vesicles. By specifying each component in advance, the bottom-up approach offers precise control over composition and function. The bottom-up approaches are discussed below.

#### Peptide-functionalized liposomes

Peptides were the earliest molecular components to be used because they allow specific functions to be installed in a modular fashion. Defined peptide sequences can promote cell-specific targeting and membrane penetration, allowing liposomes to mimic activities normally provided by vesicular surface proteins. By covalently attaching peptides to lipids or inserting them directly into pre-formed membranes, investigators produced vesicles capable of delivering specific cargos to specific cells. These systems established the central principle of bottom-up design: extracellular vesicle-like behaviors can be constructed one functional module at a time [115,116].

#### Antibody-decorated liposomes

As peptide systems matured, the field expanded to antibodies with a view to achieving even higher specificity. Antibodies can be coupled to lipids through established chemical methods or added after vesicle formation. Antibody coupling allowed vesicles to target defined cell populations and cross physiologic barriers. Dual-ligand formulations, e.g., vesicles carrying both blood–brain-barrier-penetrating peptides and tumor-specific antibodies, demonstrated how multiple targeting constraints could be engineered simultaneously [117,118].

#### Membrane-protein embedded vesicles

Although peptide and antibody decoration can promote selective binding, it does not reproduce the complexity of natural membranes found in extracellular vesicles. To address this gap, researchers began incorporating purified membrane proteins directly into synthetic bilayers. These hybrid vesicles, assembled from defined lipids and cell-derived proteins, displayed adhesion, trafficking, and immune-modulatory properties resembling those of natural extracellular vesicles. Further embedding of membrane proteins provided a route to recreate the multi-component interactions that underlie natural extracellular vesicle behavior [119,120].

#### Polysaccharide-modified vesicles

Polysaccharides introduce capabilities that lipids and peptides are unable to provide. For example, the positive charge of the polysaccharide chitosan is useful for gene delivery because it will tightly bind to nucleic acids. Similarly, incorporation of hyaluronic acid provides a highly hydrated, biocompatible surface layer that reduces nonspecific interactions as well as selectively binds to CD44-expressing cells. By modifying liposomes with these polysaccharides, designers can improve stability, enhance targeting, and load new types of cargo. The net effect is an expanded functional range for synthetic extracellular vesicle mimics [121,122].

#### Protein- and polymer-based vesicles

A more radical approach replaces lipids entirely with protein–polymer conjugates that self-assemble into membrane-like vesicles. These constructs can encapsulate macromolecules, maintain selective permeability, and even support cell-free protein synthesis. Protein components are produced recombinantly, and their sequences can be engineered in advance to adjust binding motifs, assembly behavior, or catalytic activity. Sequence-level tunability allows precise control over vesicle properties [123].

#### Fully synthetic extracellular vesicles

The most sophisticated bottom-up strategies reconstruct extracellular vesicle-like vesicles from defined lipid, protein, and nucleic acid components. By combining quantitative lipid formulations with charge-mediated assembly methods, these systems generate vesicles that closely approximate the physical composition, cargo-loading characteristics, and functional behaviors of natural extracellular vesicles. Such fully synthetic constructs demonstrate that bottom-up assembly can recapitulate not only individual extracellular vesicle activities but also coordinated, multi-component functions characteristic of native vesicles [124–126].

## Extracellular vesicle applications in the heart

Ongoing clinical research specifically investigating extracellular vesicle-based therapeutics for cardiac repair and regeneration remains limited. As of December 2025, using the search terms ‘heart’ and ‘extracellular vesicles/vesicles’, ClinicalTrials.gov lists 19 trials. Most of these trials evaluate extracellular vesicles as biomarkers of cardiac disease rather than as therapeutics. In contrast, only three registered trials are testing extracellular vesicles as interventions: (1) treatment of heart failure (NCT05669144, Phase I/II), (2) drug-refractory left ventricular dysfunction secondary to non-ischemic dilated cardiomyopathy (NCT05774509, Phase I), and (3) reduction of stent-induced myocardial injury (NCT04327635, Phase I). All three utilize native, unmodified extracellular vesicles.

Although clinical trials are few, preclinical studies are abundant. Representative studies are summarized in Table 2. Native and engineered extracellular vesicles have demonstrated improvements in multiple cardiac injury models, including myocardial infarction and ischemia–reperfusion injury, as well as pressure-overload hypertrophy. However, study designs vary substantially in dose, route, and timing. For example, in rodent models, reported dosing spans between 10 and 100 μg or 10^7^ and 10^11^ particles of protein per administration. Delivery strategies also differ widely, including intramyocardial injection, intravenous infusion, intranasal/inhalation delivery, and epicardial application. Interstudy heterogeneity, combined with inconsistent reporting of dose units (μg protein versus particle count), dosing frequency, and functional endpoints, hinders translating preclinical efficacy to the clinic [127].

**Table 2:** Preclinical data from extracellular vesicle studies in heart disease

Cargo	Extracellular vesicle source/engineering	Target/pathway	Functional outcome	Reference
siNOX4	Cardiac-targeting sEVs (blood-derived, conjugated with cardiac-targeting peptides via click chemistry)	NOX4 → MAPK, NF-κB, TGF-β/Smad	↓ Oxidative stress, ↓ hypertrophy & fibrosis, improved cardiac function	[128]
siSAV1	Adipose-derived stem cell extracellular vesicles	Hippo pathway (SAV1 silencing → YAP activation)	Cardiomyocyte cell cycle re-entry, cardiac regeneration, anti-fibrotic, pro-angiogenic	[129]
siRAGE	Cardiac-targeting sEVs (CTP-LAMP2b fusion)	RAGE → NF-κB signaling	↓ Inflammation, ↓ cytokine production, reduced cardiac injury	[130]
HSP-20	Cardiomyocyte-derived extracellular vesicles	Inhibits TNF-α and IL-1β	Cardioprotective, anti-inflammatory	[131,132]
HSP-60	Cardiomyocyte-derived extracellular vesicles	extracellular form → pro-inflammatory, atherogenic	Dual role: protective intracellular, harmful extracellular	[133,134]
HSP-70	Cardiomyocyte-derived extracellular vesicles	TLR-4 → MAPK/ERK1/2 signaling	Pro-survival, cytoprotection	[135,136]
HSP-27	Macrophage-derived extracellular vesicles (cholesterol-loaded THP-1)	Promotes cholesterol efflux	Improves cholesterol homeostasis, potential anti-atherosclerotic role	[137]
miR-320	Diabetic cardiomyocyte-derived extracellular vesicles	Angiogenesis regulators	Impaired angiogenesis	[138]
miR-21-5p	Diabetic heart extracellular vesicles	NF-κB	Modulates inflammation/fibrosis	[138]
miR-146a	Diabetic heart extracellular vesicles	TGF-β/Smad	Anti-fibrotic	[138]
miR-144-3p	CTP-EVs (curcumin + miRNA co-delivery)	Oxidative stress/apoptosis pathways	Improved bioavailability, cardioprotection	[139]
miR-210	MSC-derived extracellular vesicles	AIFM3 → PI3K/Akt	Anti-apoptotic, ↓ infarct size	[140]
miR-214	MSC-derived extracellular vesicles	PTEN → PI3K/Akt	↑ survival, ↑ angiogenesis, ↓ infarct size	[141]
miR-302	CMP-engineered cardiomyocyte-targeted extracellular vesicles	Hippo → YAP activation	↑ Proliferation, regeneration, improved function	[142]
miR-126	ELVs (engineered vesicles)	SPRED1 → PI3K/Akt	↑ Angiogenesis, improved function	[143]
miR-181b	CDC-extracellular vesicles	PKCδ (macrophages)	Reparative polarization, ↓ infarct size	[144]
miR-21	MSC-derived extracellular vesicles	PTEN/Akt	Angiogenesis, cardioprotection	[145]
miR-23a-3p	MSC-derived extracellular vesicles	PTEN/Akt	Angiogenesis	[146]
miR-30b	MSC-derived extracellular vesicles	DLL4 down-regulation	Angiogenesis	[147]
miR-130a-3p	MSC-derived extracellular vesicles	HOXA5 inhibition	Angiogenesis	[148]
miR-132	MSC-derived extracellular vesicles	RASA1 inhibition	Angiogenesis	[149]
miR-205	MSC-derived extracellular vesicles	HIF-1α, VEGF	Vascular growth	[150]
miR-210	MSC-derived extracellular vesicles	Efna3 suppression	Endothelial function, angiogenesis	[151]
miR-223-5p	MSC-derived extracellular vesicles	CCR2 signaling	↓ Monocyte infiltration, ↓ I/R injury	[152]
miR-1246	MSC-derived extracellular vesicles	PRSS23 → Snail/α-SMA	Remodeling in chronic heart failure	[153]
miR-126	MSC-derived extracellular vesicles (rat adipose)	Inflammation/angiogenesis (SPRED1, PI3K/Akt)	Reduces inflammation, promotes neovascularization	[154]
Fatty acids	Serum-derived extracellular vesicles	CD36-mediated uptake	Metabolic regulation in cardiomyocytes and endothelial cells	[155]

## Future perspectives

The rapid evolution of extracellular vesicle engineering strategies has brought us closer to their clinical translation for heart failure. However, significant challenges remain. A critical bottleneck for both native and engineered extracellular vesicles is the establishment of standardized manufacturing procedures under GMP guidelines, ensuring high yield, purity, and consistent batch-to-batch composition. Addressing the transient *in vivo* half-life and off-target accumulation (predominantly in the liver, spleen, and lungs) is another important area, requiring innovative delivery systems such as localized hydrogels or novel administration routes to maximize cardiac retention.

From a scientific standpoint, future studies will need to delve deeper into the precise mechanisms through which these highly engineered extracellular vesicles modulate the complex cardiac microenvironment, including their interactions with immune cells and cardiac fibroblasts, to ensure efficacy and long-term safety. The integration of emerging approaches, such as the application of MBVs within engineered cardiac patches or scaffolds, offers exciting potential for sustained, localized therapeutic effects that mimic natural tissue repair and moving beyond single-injection systemic therapies.

Finally, moving toward the clinic necessitates clear regulatory pathways and large-scale, multicenter trials to confirm therapeutic efficacy across diverse patient populations, ultimately bridging the gap from promising preclinical results to approved precision therapeutics in cardiovascular medicine.

## Abbreviations

α-SMA: α-smooth muscle actin
BFC: bifunctional chelators
CTP: cardiac targeting peptide
ECM: extracellular matrix
EGFR: epidermal growth factor receptor
EPCs: endothelial progenitor cells
GMP: good manufacturing practice
GPIs: glycoinositol phospholipids
HIF-1α: hypoxia inducible factor-1α
LAMP-2B: lysosome-associated membrane protein 2B
MBVs: matrix-bound extracellular vesicles
MSCs: mesenchymal stem cells
PDGFR: platelet-derived growth factor receptor
PEG: polyethylene glycol
SPAAC: strain-promoted azide–alkyne cycloaddition
